# Basal Levels of CD18 Antigen Presenting Cells in Cow Milk Associate with Copy Number Variation of Fc Gamma Receptors

**DOI:** 10.3390/genes11080952

**Published:** 2020-08-18

**Authors:** Eyal Seroussi, Shlomo E. Blum, Oleg Krifucks, Andrey Shirak, Shamay Jacoby, Gabriel Leitner

**Affiliations:** 1 Institute of Animal Science, ARO, Volcani Center, HaMaccabim Road, P.O. Box 15159, Rishon LeTsiyon 7528809, Israel; shiraka@volcani.agri.gov.il (A.S.); shamijac@gmail.com (S.J.); 2 National Mastitis Reference Center, Kimron Veterinary Institute, P.O. Box 12, Bet Dagan 50250, Israel; shlomobl@moag.gov.il (S.E.B.); olegkr@moag.gov.il (O.K.); leitnerg1@gmail.com (G.L.)

**Keywords:** SNP BeadChip, cluster of differentiation, classification determinant, GWAS, milk, mastitis, immunogenetics

## Abstract

Differentiation of cells by flow cytometry provides informative somatic cell counts (SCCs) that allow analyzing leukocyte population patterns in udder infections of different etiologies. Postulating that this approach also enhances the statistical power to detect genetic variants linked to cell levels in milk of healthy mammary glands, we used monoclonal antibodies anti-CD18, anti-CD4, anti--CD14, and anti-PMN to count cells presenting these surface antigens, and performed a genome-wide association study of these counts in 125 Israeli Holsteins genotyped using SNP BeadChips. We identified an informative haplotype of 15 SNPs in the centromeric end of BTA3 that was strongly associated with CD18 cells (*p* < 2.3 × 10^−9^). Within this region, examination of the network of genes interacting with *ITGB2* (CD18) indicated an Fc-γ-receptor gene cluster, including *FCGR2A* (CD32). Sanger-sequence analysis of *FCGR2*s-linked exon 3 variation to CD18 counts. Meta-analysis of RNA-Seq data revealed a significant negative correlation (R = −0.51) between expression of CD32 and CD18 in milk. Assembly of DNA-Seq reads uncovered FCGR copy-number variation and a variant, designated *V7*, was abundant in dairy cattle, probably reflecting adaptation to selection pressure for low SCC in Holstein milk.

## 1. Introduction

Mastitis—inflammation of the mammary gland—results in the most significant economic losses to the dairy industry worldwide [1]. Incidents of mastitis are numerous and can reach up to 75% of the herd in developing countries [2]. Its cost involves drugs, labor, discarded and shorter shelf-life milk, reduced cheese yield, lower price paid for lower milk quality, and increased culling. Cows that are culled or removed from the herd are mostly replaced by first-calf heifers. Lameness, disease, clinical and subclinical mastitis, and mortality force involuntary herd management and disrupt the voluntary management plan of culling old, genetically and/or physiologically low-producing and infertile cows.

Bacteria are the major causative pathogens of mastitis. They are distributed all over the cow’s skin, including the external part of the streak canal, and are part of the flora of the cow’s environment. However, regardless of environmental conditions and management, many cows/glands are not inflamed by mastitis, indicating that genetics may play an important role. Penetration of bacteria into the mammary gland, mostly through the teat canal, is crucial for the development of mastitis. However, not every bacterium passing through the teat canal will end up causing mastitis. Early elimination of the bacteria by innate immune factors such as phagocytes makes it impossible to calculate the percentage of bacterial elimination. What can be evaluated is the distribution of animals with identified clinical and/or sub-clinical mastitis in the entire population. However, unlike the clinical form, determination of sub-clinical mastitis is problematic due to a lack of visual symptoms. Thus, in most studies related to sub-clinical mastitis, mastitis detection at cow or quarter levels is based on the California mastitis test (CMT) and somatic cell count (SCC), sometimes together with bacteriology (for more details, see [3]). In large genetic analyses of mastitis cases, the major and sometimes only indicator of sub-clinical mastitis is SCC, which is determined monthly or more frequently. However, the relation of these data to mastitis lacks sensitivity and details: (1) in most cases, only one gland is infected and therefore cow SCC is low (<100,000 cells/mL); moreover, in many studies, the threshold of 200,000 cells/mL for a healthy cow is considered; (2) in some clinical cases, the cow is treated and the SCC decreases within days to <200,000 cells/mL; therefore, if the infection occurs between two routine monthly milk recordings, the incidence of mastitis may go unnoticed, unless it is recorded by hand; (3) in many cases, a bacteriology test is not performed and even when it is, identification is at the species level. Therefore, the data for analysis, if recorded, include the severity and duration of the inflammation induced by bacteria, the host’s immunological response, and their interaction. Important parameters of this immunological response are the counts of leukocytes in the host’s milk [4,5]. In this work, we analyzed the association between the genomes of 148 dairy heifers and their udder health with milk cells expressing common cluster of differentiation (CD) antigens, which are cell-surface molecules that provide targets for the immunophenotyping of leukocytes.

## 2. Materials and Methods

### 2.1. Animals and Experimental Scheme

The study was conducted in one herd of ~200 Israeli Holstein dairy cows producing an average milk yield of >11,500 L/305 lactation days. All treatment protocols were approved by the Institutional Animal Care Committee of the Agricultural Research Organization, Volcani Center, Bet Dagan, Israel (# 16_b7736_10). The dairy parlor has been previously described [6]. Briefly, it is equipped with a computerized AfiFarm herd-management system and AfiLab milk analyzer, providing on-line data on gross milk composition and conductivity. Cows were milked thrice daily at 04:00, 13:00, 20:00 h. Routine monthly milk yield, milk composition, and SCC were recorded by the Israeli Cattle Breeders Association (ICBA, Caesarea, Israel). Gross milk composition analysis, including fat, protein, and lactose contents, was performed with a Milkoscan FT+. SCCs were performed with a Fossomatic FC (Foss Electric, Hilleröd, Denmark). Seventy to eighty percent of female calves are raised on farms for replacement of older cows. Female calves were raised in groups of similar age, artificially inseminated at 13–15 months and transferred to a parturition yard 3 weeks before expected delivery. Post-calving, heifers were relocated to a first-calf heifer group through first lactation. In 2015–2017, all heifers from calving to dry-off entered the study. Every abnormality, with an emphasis on the udder, was recorded and tested by the researcher for infection by CMT on the individual gland, and samples for bacteriology were taken when CMT results were >1 (range: 0, trace, 1, 2, 3). The same procedure was conducted for each of the heifers with SCC above 80,000 cells/mL, following the routine monthly milk-recordings by the ICBA. When diagnoses revealed bacteria and/or only inflammation, two more samples were taken 2–3 days apart. At 70–120 days in milk, at peak lactation, heifers were tested (each quarter) for bacterial infection and milk composition, including whether or not SCC was elevated or abnormalities were present. After verification of each quarter’s condition, composite milk of all non-inflamed glands (no bacteria and SCC <50,000 cells/mL) was sampled during the afternoon milking and sent for analyses of milk composition, SCC, and differential SSC. Milk bacteriology identification was conducted according to the International Dairy Federation [7]. Briefly, 10 µL of each milk sample was inoculated onto blood agar (enriched with 5% washed sheep red blood cells) and MacConkey agar plates (Bacto-Agar, Difco Laboratory, Becton Dickinson, Le Pont de Claix, France). Plates were incubated at 37 °C and examined for bacterial growth after 18 and 42 h. General data of date of parturition, daily and lactation milk yield, milk composition, number of artificial inseminations (AIs) to pregnancy, length of lactation, medical events, and culling were recorded from the on-line and ICBA Herd Book. Clinical mastitis and some of the sub-clinical mastitis cases were treated with antibiotics.

### 2.2. Antibodies, Conjugates, and Differential SCC

Somatic cell distributions were tested by flow cytometry (FACs Calibur, Becton-Dickinson, San Jose, CA, USA) as described previously [8]. Briefly, milk samples were analyzed not more than 3 h after collection. The number of somatic cells in each sample was determined by cell counter (Coulter Electronics Ltd., Beds, England) and the volume of milk containing approximately 1 × 10^6^ cells was centrifuged (10 min, 230 g, 4 °C), pellets were resuspended in 15 mL phosphate-buffered saline (PBS) and washed by centrifugation (5 min, 200 g, 4 °C). The washed pellets were resuspended in 0.2 mL PBS with a single monoclonal antibody (mAb) or with a pair of mAbs, incubated (1 h at 4 °C), washed (×3) in PBS, and reacted with the conjugated secondary antibodies (30 min, 4 °C). Following incubation, the cells were washed and resuspended in 1 mL PBS. To calculate the percentages of the different leukocytes 10,000 events were read per sample. The commercial mAbs (Kingfisher Biotech Inc. Saint Paul, MN, USA) used for the detection of different leukocytes were: (i) leukocytes, anti--CD18: BAQ30A (IgG-1, RRID:AB_2745264); (ii) lymphocytes, anti--CD4: GC50A1 (IgM, RRID:AB_2848215); (iii) T-cytotoxic lymphocytes, anti-CD8: CACT80C (IgG1, RRID:AB_2848216); (iv) monocytes/macrophages, anti--CD14: CAM36A (IgG1, RRID:AB_2745247); (v) polymorphonuclear (PMN) leukocytes, anti-PMN: CH138A (IgM, RRID:AB_2857323). Paired analyses were performed with anti-CD18 and anti-PMN or anti-CD14 mAbs. All mAbs were species--reactive with bovine cells. Secondary polyclonal antibodies were goat F(ab’)2 anti--mouse immunoglobulin IgM (μ-chain specific) conjugated with fluorescein isothiocyanate (FITC) that exhibited minimal cross--reaction to human--, bovine--, and horse--serum proteins (Jackson Immuno Research Lab., West Grove, PA, USA) and affinity isolated goat anti--mouse IgG--1 conjugated with TRI--COLOR (CALTAG Laboratories, Burlingame, CA, USA). The counts of cells presenting these five CD antigens are presented as percentages of the average basal SCC of a healthy gland (44 × 10^3^ cells/mL) in Supplementary File S1.

### 2.3. The Dataset, Haplotype Phasing, and Trait-Association Analysis

Seven traits were analyzed; counts of leukocytes presenting antigens i, ii, iii, iv, v, mean lactation somatic cell score, and infection status. Hairs with roots were pulled from the cows’ tails and submitted for Illumina (San Diego, CA, USA) BovineSNP BeadChip genotyping. Association for bovine genome single-nucleotide polymorphisms (SNPs) was initially determined using PLINK [9], activating the haplotype sliding-window options (--cow --maf 0.05 --hwe 0.0001 --geno 0.1 --hap-window 15 --hap-linear --hap-omnibus). A haplotype near BTA3’s centromere, consisting of 15 SNPs (ARS-BFGL-NGS-30351, ARS-BFGL-NGS-97347, ARS-BFGL-NGS-113990, ARS-BFGL-NGS-8587, ARS-BFGL-NGS-2694, ARS-BFGL-NGS-27948, ARS-BFGL-NGS-6957, BTA-69900-no-rs, ARS-BFGL-NGS-70744, ARS-BFGL-NGS-25382, Hapmap40339-BTA-117016, ARS-BFGL-NGS-87298, ARS-BFGL-NGS-23250, Hapmap49050-BTA-119760, ARS-BFGL-NGS-33062) within nucleotide positions 8,660,956–11,117,776 (build ARS-UCD1.2), was chosen for further analyses. Association for these SNPs was determined using sliding-window PLINK analysis with bootstrapping option (--hap-window 15 --hap-linear --mperm 200000).

### 2.4. FCGR2 Exon 3 Sequencing and Peak Height Analysis

Template DNA was amplified using PCR primer pair #1 of bovine FCGR2 genes: forward 5′-TCTGAGATTTGGGGTCTGCT-3′ and reverse 5′-GCAGATTTCATCTCCCCTTG-3′ designed in the second and third introns, respectively. Amplification of these 384-bp amplicons was performed using the Bio-X-ACT™ Long Kit (Bioline Ltd., London, UK) according to the manufacturer’s instructions and the following conditions: 30 cycles for 40 s at 92 °C, 60 s at 63 °C, and 60 s at 68 °C. In the current genome build (ARS-UCD1.2), these amplicons match BTA3 nucleotide positions 7,942,495–7,942,878 and 8,040,791–8,041,174, within genes *FCGR2B* and *FCGR2A*, respectively.

PCR products were separated on agarose gels, excised, purified with AccuPrep^®^ Gel Purification Kit (BioNeer Corp., Seoul, Korea), and then sequenced from both directions using an ABI3730 sequencer and primer pair #1. PCR primers were designed using Primer3 [10]. Nucleotide height or area ratios for the double peaks observed in the ABI trace files were the ratios of the peak values obtained from the poly file generated by Phred base caller [11], activating the write poly file options (phred –d).

### 2.5. Sequence Read Archive (SRA) Search

SRA BLASTN searches were performed using the dedicated NCBI server [12]. This search was conducted using probes (Table S1) with word size of 32 bp and the Megablast default setting optimized for detection of highly similar sequences. The normalized unit of transcript expression of Reads Per Kilobase of transcript, per Million mapped reads (RPKM) was calculated by 1,000 × (Number of BLAST hits/32) × (10^6^/Total mappable reads).

### 2.6. Deep Sequencing and Analysis of Bovine Genomes

The current reference genome is based on the Hereford beef breed. To find variations between the dairy and beef breeds that may underlie the differences in *FCGR2*s, DNA was extracted from thawed frozen semen of a single Holstein AI sire (JJ, HOLISRM000000007424, BioSample: SAMEA5524117) and was deep-sequenced using the next-generation sequencing platform of Illumina HiSeq2000 according to the manufacturer’s paired-end protocol. Average fragment length was 580 bp, and 100-bp sequence reads were obtained from both ends. DNA sample was applied to two lanes, yielding ~30-fold (906,996,192 reads) coverage for this sample. The reference gene sequence was then used as a template for mapping these DNA-Seq reads using GAP5 software [13]. BWA options for this mapping were set to bam bwasw -t 8 -T 60 [14]. The assembled sequence of this sire gene was submitted to ENA under project ID PRJEB38396, ENA accession nos. ERX4137343 (BAM format), and LR798693-6 (annotated gene sequence).

### 2.7. Statistical Analysis

The genetic correlations among traits or among markers’ substitution effects were estimated as Pearson’s correlation coefficients. These coefficients of correlation were calculated using R package [15] or CORREL function in Excel spreadsheet (Microsoft Corporation, Santa Rosa, CA, USA), respectively. The significance of the correlation coefficient (R), at confidence level of 95% of n observed samples was confirmed by a t-test using the formula: t-value = R × (n−2)^0.5^/(1−R^2^)^0.5^.

## 3. Results

Aiming for better management of udder health, we recorded immunological, genomic and production parameters of 148 first-calf heifers at the average age of 24 months (Supplementary File S1). In addition to the infection status of each quarter gland, immunological parameters included cytometry counts for five CD markers of milk leukocytes sampled from a heathy gland. These are presented as percentages of the average basal SCC of the healthy gland, which was 44 × 10^3^ cells/mL (SE ± 2.5 × 10^3^, *n* = 116). Genomic data consisted of Illumina BeadChip SNP genotypes, and production records were the average values obtained for milk, fat, protein, and SCC at first lactation. According to infection status, we divided the heifers into three groups: uninfected, and clinically and sub-clinically infected; 65 heifers (43.9%) did not have mastitis and mean lactation SCC was 55 × 10^3^ cells/mL (SE ± 3 × 10^3^, *n* = 63), and 82 heifers (55.8%) had sub-clinical or clinical infection in one or more glands. With first-lactation average SCCs of 263 ± 35 × 10^3^ and of 562 ± 114 × 10^3^ in the sub-clinical (*n* = 58) and clinical (*n* = 24) groups, respectively, these values significantly differed among the groups (*p* < 0.001). No significant differences were found between the heifer groups with respect to basal CD marker counts in the healthy mammary glands.

### 3.1. Genome-Wide Association Study (GWAS) of Immunological Traits

**Figure 1:**
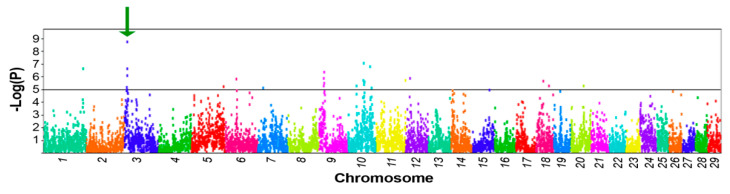
Genome-wide association analysis of basal CD18 leukocyte counts. Omnibus *p*-values for 15-SNP haplotype association were determined using PLINK. Green arrow points to the most probable association. Black horizontal line denotes the threshold value of significance considering Bonferroni correction for multiple comparisons.

Considering adjustments for multiple comparisons and population stratification, we also did not observe any significant association between SNP genotypes and heifer infection status or SCC. However, using PLINK software [9] sliding-window analysis over the bovine genome, we identified an informative haplotype of 15 SNPs in the centromeric end of BTA3 that was strongly associated with the CD18 leukocyte counts (*p* < 2.3 × 10^−9^, Figure 1). The identified haplotype consisted of SNPs on the BovineSNP50K BeadChip at positions 9–11 Mb (build ARS-UCD1.2). The PLINK permutation option was employed to further verify the probability of association of the haplotype alleles with CD18 leukocyte counts. For this window, 24 common haplotypes explained >74% of the observed sequence variation (Table 1). Sorting the haplotypes by frequency, the likelihood of association with CD18 leukocyte counts was significant only for presence/absence of the fourth haplotype (#4, F = 5%), which associated with higher counts (β-value 11,600 cells/mL, Table 1). Since this simplified analysis may be confounded by population stratification and multiple comparisons, we applied bootstrapping with 200,000 permutations, which corroborated the significance of this association (EMP2 < 10^−5^, Table 1). It should be noted that the CD18 counts were highly correlated (R = 0.93) with the PMN cell counts and thus mainly represented the basal level of PMN leukocytes in a healthy mammary gland.

### 3.2. Analysis of Candidate Genes

**Figure 2:**
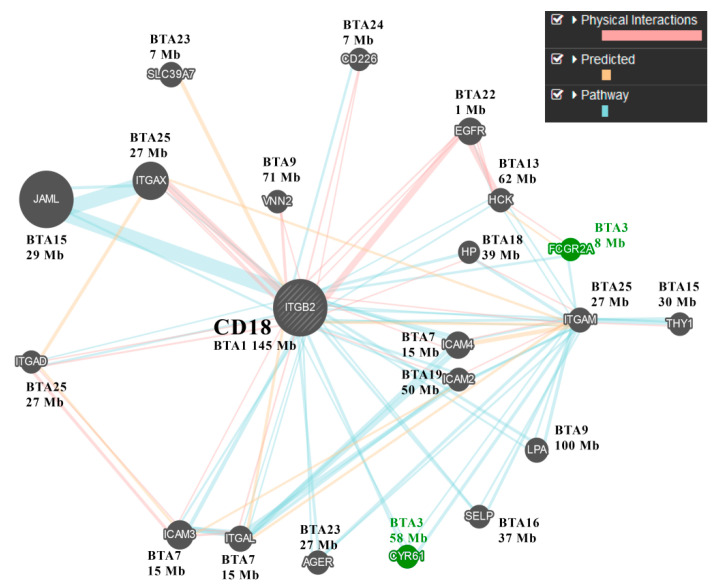
Gene network of the *ITGB2* (CD18) gene. Connections between nodes were based on protein–protein interaction data, predicted functional relationships between genes, and gene-pathway data produced by the GeneMANIA system using *ITGB2* as the input gene and the *Homo sapiens* database. On the output of this prediction server, next to the gene nodes, the orthologous bovine autosome numbers and positions were annotated. The nodes of genes with positions coinciding with BTA3 are highlighted in green.

**Figure 3:**
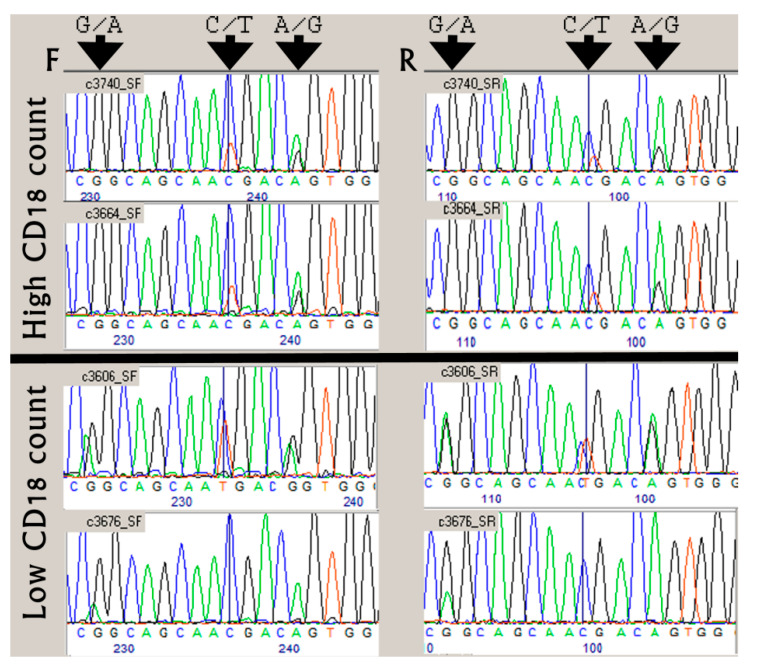
Sequence chromatograms of polymorphic stretch in CD32 third exon of high or low milk CD18-expressing heifers. Tail hairs of high (top) and low (bottom) CD18 leukocyte-count heifers were collected and the extracted DNA was used for simultaneous PCR amplification of the third exons encoding CD32. Forward (F) and reverse (R) orientation chromatograms show three variations (indicated by arrows) in which the ratio of nucleotide heights provides information on the number of paralogs encoding different antigen configurations. In these positions, G-C-A and G-T-G represent the reference sequences of FCGR paralogs *2B* and *2A*, transcript IDs: NM_174539 and NM_001109806, respectively.

Although the gene encoding CD18 (*ITGB2*) is mapped to the telomeric end of BTA1, the effect on counts of CD18 antigen-presenting cells in milk associated with this locus (T-Stat = 72.3, 15-SNP haplotype between SNPs ARS-BFGL-NGS-4848 and ARS-BFGL-NGS-86458) was much weaker than that recorded for the centromeric end of BTA3 (T-Stat = 90.6, Figure 1). Apparently, in our heifer sample, genetic variation encoded by a BTA3 gene had the major effect on counts of CD18 antigen-presenting cells. To locate this gene, we examined the network of genes that are likely to interact with *ITGB2* (Figure 2). This analysis pointed to two genes on BTA3; one of them, *FCGR2A* (CD32), is a member of the Fc γ receptor (FCGR) gene cluster. This cluster is located at the centromeric position (8 Mb, Figure 2), similar to the SNP haplotype that had the maximal effect on CD18 leukocyte counts. Indeed, in humans, the FCGR gene cluster has long been associated with frequent genetic polymorphism, including copy-number variations (CNVs) that are involved in modulating important immune functions [16]. As we noticed four recorded variations between the reference sequences of the third exons of bovine *FCGR2s* (NM_001109806; NM_174539), we simultaneously tested whether this genetic variation associates with counts of CD18 antigen-presenting cells, by Sanger sequencing of the third exon (Ex3) of the *FCGR2A* and *2B* genes using PCR primers designed at the adjacent introns. Comparing sequencing chromatograms of high (haplotype #4 carriers) and low CD18-expressing heifers revealed the abundance of the genetic variation encoded by this exon, including a polymorphic 13-bp stretch with three nucleotide variations (Figure 3). Sequence chromatograms (*n* = 15) of the CD32-encoding region showed that heifers with high CD18 counts (*n* = 4) had a 1:2 copy ratio between antigen genes *2B* and *2A*, whereas the others exhibited the 1:1 or 0:1 pattern (*p* < 0.03, Figure 3). Moreover, at the first variable position (G/A, Figure 3), only low-expressing heifers carried the adenine nucleotide, which did not match any reference sequence for FCGR genes, suggesting that this common novel variant may drive low CD18 counts of leukocytes in milk. At this position, most non-carriers of haplotype #4 displayed either 1:1 or 1:2 copy ratios between paralogs (Figure 3).

### 3.3. CD32 vs. CD18 Gene Expression

Following the indications of genomic variation with functional effect in the bovine *FCGR2* genes, we examined whether this variation is transcribed and if current reference sequences of the *FCGR2* genes that have been derived from beef cattle also match their repertoire in Holstein cattle. Using 32-bp word BLASTN search (Supplementary Table S1), milk RNA-Seq submissions of 100 Holstein cows deposited in the SRA (project ID PRJNA305942) were searched for the eight possible haplotypes of the polymorphic 13-bp stretch in *FCGR2* Ex3 and for the *ITGB2* sequence spanning the splice junction of the second and third exons (Supplementary Table S2). Of these probes, probe 9 matching *ITGB2* was most frequently expressed, while *FCGR2B* (probe 4) was frequently detected, and *FCGR2A* (probe 1) was rarely expressed (Supplementary Table S2). Besides the frequent expression of an unknown *FCGR2* variant (probe 7), other probes were not detected or detected only sporadically (Supplementary Table S2). Within this sample, the total read counts varied widely between 60 thousand and 45 million, suggesting that technical factors influenced the quality of the results (Table S2). To further analyze the results, we selected samples with at least 200 probe hits, for which we extended our BLASTN search to RNA-Seq data derived from blood (18 cows, Table 2). The few cows (*n =* 5) that did not express the unknown *FCGR2* variant (probe 7) in milk were the only ones that did not express it in the blood, suggesting that lack of expression emerged from genomic variation. Otherwise, total read numbers and RPKM values showed no correlation between the expression values in milk and in blood. Nevertheless, a significant negative correlation (R = −0.51, *p* < 0.03) was observed between expression of CD32 and CD18 in milk; and in certain sections, the sample negative R values peaked at over 0.8, as exemplified by sliding-window correlation analysis (Table 2). This correlation supported the hypothesis that genetic variation in the CD32 gene influences CD18 leukocyte counts in milk but not in blood, which is expected since leukocytes migration into the mammary gland is not likely to have a pronounced effect on the blood reservoir of circulating leukocyte.

### 3.4. Computerized Cloning of FCGR Genes of an Influential Israeli Holstein Sire

**Figure 4:**
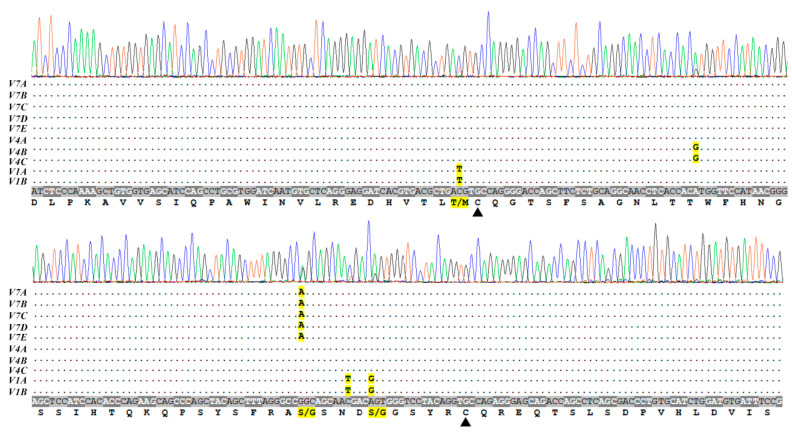
Sequence chromatograms of CD32 third exon of a representative Holstein sire compared to an allele model based on DNA-Seq. DNA extracted from semen was used for simultaneous PCR amplification of the third exons encoding CD32. Chromatograms show five variations in which the ratio of nucleotide heights provided information on the number of encoding paralogs. Below the chromatogram, dots indicate similarity to the consensus sequence of 10 allele variants predicted by the assembled sequences and counts of this sire’s DNA-Seq reads. Putative amino acid translation is given below the consensus sequence, in which codons are annotated by alternating font and background color. Nucleotide and amino acid variations are highlighted in yellow. Arrowheads point to the two cysteines predicted to form the disulfide bond that stabilizes an extracellular structure of the immunoglobulin-like C-2 type.

Since the sequence of the unknown *FCGR2* variant (probe 7) matched the novel variant detected by Sanger sequencing (adenine nucleotide in the position of the first variation, Figure 3), we chose to further characterize this transcript, which was abundant in Holstein cattle and thus may reflect adaptation to selection pressure toward low leukocyte counts in Holstein milk induced by the breeding program. We assembled expressed sequence tags (ESTs) of four clones (EST IDs: FE019948-9, FE020120-1, EE937361-2, and BE845572), realizing that this transcript was similar to *FCGR2B* (GenBank ID: NM_174539) except for a missense variation resulting in a non-conservative serine-to-glycine substitution in the predicted extracellular structure of the immunoglobulin-like C-2 type, which is encoded by Ex3 (Figure 4).

Using the 32-bp word BLASTN search, we meta-analyzed genomic DNA-Seq submissions (SRA, project ID PRJNA343262) of 20 dairy (Holstein) and 20 beef (Angus, Hereford, Charolaise, Limousine, Simmental, four individuals each) cattle. The number of hits for the eight possible haplotypes of the polymorphic 13-bp stretch in *FCGR2* Ex3 was compared to those for three control genes (*HOXB7*, *HOXA7*, *SRY*), for which CNV is assumed to be rare (Supplementary Table S2). The ratio of hits for probe 7 and total hits for *FCGR2* was indeed significantly higher for Holsteins (24%, *p* < 0.01) than for beef cattle (9%). Nevertheless, while this probe was not detected in the Hereford and Limousine individuals, it was common in the others (e.g., Simmental, 21%). *FCGR2* copy numbers based on correction for coverage and average detection rate of the control gene probes fluctuated between two and nine (Supplementary Table S2).

To better study the *FCGR2* gene sequence and copy number in dairy cattle, we analyzed a representative Holstein sire (JJ, HOLISRM000000007424), applying deep sequencing to the genome. At the end of 2018, this sire was recorded in the top 20 sires for total net merit, with more than 10,000 daughters. Being a descendant of the popular US bull O--Bee Manfred Justice (HOUSA000122358313), this sire represents an influential bloodline of Holstein cattle. Directed assembly was hampered by the presence of multiple similar gene copies. Focusing on the most polymorphic third exon, at least four distinct alleles were detected (Figure 4). Following our eight probe sequences in *FCGR2* Ex3, we named these alleles/variants using the prefix “V” (i.e., *V1a*, *V1b*, *V4a*, *V4b*, *V4c,* and *V7*). For each of the six contigs, relative copy number was estimated based on the number of reads that spanned the central variation in the polymorphic 13-bp stretch in *FCGR2* Ex3. The count in the *V7* contig (*n* = 28) was similar to the combined count of the other contigs (*n* = 25, consisting of *V1a*, *V1b*, *V4a*, *V4b,* and *V4c* with 7, 3, 2, 11, and 2 reads, respectively). This suggested that at least five copies, designated *V7a*––*V7e*, were present in this sire genome. To further support this hypothesis, we performed peak-height analysis of Sanger chromatograms obtained for the Ex3 amplicon. Indeed, the adenine residue typical of *V7* displayed equal peak heights compared to the guanine base of the other variants (Figure 4). In the other four polymorphic positions, peak heights also well fitted the model described in Figure 4, in which 10 allele copies of Ex3 were co-sequenced, resulting in the observed superimposed pattern. Thus, the examined sire had a CNV with at least five *FCGR2* genes, compared to the two genes in the bovine reference genome. Moreover, the number of *V7* allele copies (*n* = 5) indicated that it could have arisen from an independent paralog, which we named *FCGR2C* (ENA accession no. LR798694). Although the transcript of the *FCGR2C* variant was almost identical to that of *FCGR2B*, the introns in the assembled *FCGR2C* contig were more similar to those of *FCGR2A* (Experiment accession no. ERX4137343).

## 4. Discussion

SCC in cows’ milk is a useful indicator of milk quality and of mastitis, the costliest disease in dairy production. Understanding the genetic architecture of immune traits is essential to combating mastitis, and SCC is the most widely used trait in GWASs that aim to examine this architecture. However, in a typical mammalian genome, about one-tenth of the genes with recorded GO annotation for biological process are of immune system processes, e.g., in humans, 3,263/34,673 [17]. This abundance of inflammatory and immunological genes hinders GWASs, which are likely to falsely map “ghost” QTLs that arise due to the superposition of additive and epistatic polygenic effects near chromosomal regions that are rich in immune system genes. Albeit at the cost of a loss in power, statistical methods can control for another type of false-positive QTL, which occurs in structured populations; the “ghost” QTL problem is further complicated by such loci that are in linkage disequilibrium with a causal variant [18]. These complications may explain why in general, the overlap in genome-mapping results on QTLs affecting clinical mastitis and somatic cell score is low, even in Nordic countries where the breeding system is best at recording mastitis [19]. Knowledge of the proportion of somatic cell types in milk, rather than just the overall SCC, provides valuable information on the inflammatory status of the udder, and differential SCC has heritability (0.08) that is double that of traditional SCCs [20]. Indeed, when controlling for multiple comparisons and population stratification, the association between SNP genotypes and heifer infection status or conventional SCC was insignificant for our sample, whereas the centromeric end of BTA3 was highly associated with the differential SCC obtained by flow cytometry for leukocytes presenting CD18. To the best of our knowledge, this is the first report of a GWAS of differential SCCs based on flow cytometry of CD antigens in milk. It should be noted that CD18 has been previously associated with leukocyte performance as a result of bovine leukocyte adhesion deficiency (BLAD), which is characterized by a lack of CD11/CD18 adhesion molecules on the leukocyte surface. This syndrome is not likely to affect our sample’s heifers as they were sired by bulls that are screened against BLAD.

Searching for a CD18-effector gene in the centromeric end of BTA3, we noticed an FCGR gene cluster, including *FCGR2A* (CD32), which belongs to the network of genes interacting with *ITGB2* (CD18) [21]. Known for CNV of FCGR genes, the orthologous cluster on HSA1 likely evolved through tandem duplication of type II and type III low-affinity FCGRs, in which a heat-shock protein family A gene (*HSP70*) is embedded [22]. Corroborating this synteny, bovine *HSPA6* is positioned between *FCGR2A* and *FCGR3A* in the current *Bos taurus* genome build (ARS-UCD1). Further support for the candidacy of *FCGR2*s as genes involved in the regulation of CD18 SCC was provided by the Sanger-sequence analysis of amplicons derived from their polymorphic third exons. This co-amplification yielded chromatogram patterns that differed between cows with high and low CD18 SCCs and thus significantly linked CD18 counts to nucleotide variation in CD32 (*p* < 0.03). A polymorphic 13-bp stretch in *FCGR2* Ex3 with three adjacent SNPs proved useful for tagging *FCGR2* variants, and out of the eight possible nucleotide combinations, using BLASTN searches of public nucleotide databases, we observed three variants (tags *v1*, *v4,* and *v7*). The bovine reference genome is based on Hereford beef cattle and lacks essential comparative information for other breeds [23] and for *FCGR2*s, the annotation includes *FCGR2A* (Gene ID: 782652) and *FCGR2B* (Gene ID: 282229), which correspond to *v1* and *v4*, respectively. This annotation is compatible with the absence of the v7 variant in Hereford individuals and the indication of *FCGR2* CNV based on comparisons of read counts between *FCGR2* tags and control genes (Supplementary Table S3). This method has previously been used to discover CNV in bovine *FCGR2B* (chr3:8,648,846–8,665,349, Table_S7_CNVR_RefSeq) [24].

Calculating peak--height ratios between SNP alleles in sequencing chromatograms is another practical method for estimating their copy number proportions, especially when similar ratios are obtained by sequencing from the forward and reverse orientations [25,26]. To better characterize *v7*, which was common in Holsteins (75% of individuals, Supplementary Table S3), we compared the sequence assembly of *FCGR2* Ex3 based on next-generation sequencing to the Sanger-sequencing chromatograms of this exon from an influential Israeli AI sire. Results of this analysis were compatible with a model of five paralogous *FCGR2*s and expansion of the *v7* paralog, which was designated *FCGR2C*. This polymorphic nature and the recent burst of duplication and divergence of the bovine *FCGR2C* are typical of immune response genes under selective pressure [27]. Such pressure can be exerted by Holstein breeding programs that select for lower SCC [28].

Seeking further confirmation of the hypothesized CD18–CD32 gene interplay, we meta-analyzed RNA-Seq data, comparing the expression levels of these antigens. This revealed a significant negative correlation (R = −0.51) between the expression of CD32 and CD18 in milk. Thus, it is possible that extra gene copies of CD32 enhance its expression and induce immunomodulation towards low CD18 SCC. In uninfected glands, leukocytes are incorrectly thought to represent the majority of the somatic cells, with a few cells that are shed from the epithelium [28]. Yet, in these glands, epithelial cells accounted for ~50% of the SCC in cows [29,30]. Therefore, using flow cytometry to detect surface CD18 may be crucial for analyses of leukocytes in basal SCCs, as CD18 is a general marker of these cells and is essential for their passage into milk [31]. Controversy exists about whether low milk cell counts increase susceptibility to mastitis [28]. We observed no correlation between mastitis and basal SCC, supporting the conclusion of [28] that a low SCC resulting from selection has no negative effect on susceptibility to mastitis. Considering the current index for selection, this scenario clearly favors individuals with a genetic architecture that supports low basal SCC combined with efficient reactivity of the healthy glands to infection. Thus, the increased copy number of *FCGR2*s and abundance of *FCGR2C* in dairy cattle probably reflect adaptation to the selection pressure towards low SCC in Holstein milk.

## Figures and Tables

**Table 1:** Association of BTA3 haplotype alleles with CD18 leukocyte counts ^1^.

#	HAPLOTYPE	F ^2^	β	STAT	P	EMP1	EMP2
**1**	GGTGAGAACGAAGGG	0.08	−1.17 × 10^3^	0.46	0.499	0.504	1.00
**2**	AAAGGAGAAGGGAAG	0.07	−2.17 × 10^3^	1.66	0.200	0.199	1.00
**3**	AGTGAGAAAAGAAGA	0.06	−2.25 × 10^3^	1.17	0.281	0.282	1.00
**4**	GGTGGGGGAGGAAAG	0.05	1.16 × 10^4^	36.00	2.54 × 10^−8^	5.00 × 10^−6^	1.00 × 10^−5^
**5**	GGTGAGAGCAAGAAG	0.04	2.11 × 10^3^	0.58	0.447	0.454	1.00
**6**	GGTGAGAAAGGAAGG	0.04	−593	0.08	0.780	0.781	1.00
**7**	GGTAAGAAAGAAAAG	0.03	−4.07 × 10^3^	1.68	0.198	0.198	1.00
**8**	GGTGAGGGCGAGAAG	0.03	−375	0.03	0.871	0.872	1.00
**9**	GGTAGAAGAAAGAGG	0.03	4.91 × 10^3^	2.06	0.154	0.150	0.98
**10**	AGTGAGAAAGGGAAG	0.03	−550	0.04	0.849	0.850	1.00
**11**	AAAAGAAGCGGAAGG	0.03	1.97 × 10^3^	0.39	0.534	0.541	1.00
**12**	AGTAGGGGAAGAAAG	0.03	−2.25 × 10^3^	0.34	0.559	0.572	1.00
**13**	AGTAGAAGCGGAAGG	0.03	−1.99 × 10^3^	0.33	0.564	0.576	1.00
**14**	GGTGGAAAAGGAAGG	0.03	3.50 × 10^3^	0.63	0.429	0.449	1.00
**15**	GGTGAGAACAAGGGG	0.02	−3.13 × 10^3^	1.33	0.251	0.253	1.00
**16**	AGTGGAAAAGAAAGG	0.02	5.67 × 10^3^	3.83	0.053	0.049	0.70
**17**	GGTGAGAAAAGAAGA	0.02	−4.01 × 10^3^	1.97	0.163	0.162	0.99
**18**	AGAGAAGACGAAAAG	0.02	−428	0.02	0.902	0.905	1.00
**19**	AGAGAAGACGAAGGG	0.01	−1.53 × 10^3^	0.20	0.658	0.668	1.00
**20**	GAAAAGAACAAGGGG	0.01	−864	0.03	0.873	0.898	1.00
**21**	GGTGAGAGAAAGGAG	0.01	3.57 × 10^3^	0.87	0.353	0.365	1.00
**22**	GGTAGAAAAGGAAAG	0.01	480	0.01	0.929	0.944	1.00
**23**	GGTGAGAACGAAAAG	0.01	−1.45 × 10^3^	0.14	0.706	0.716	1.00
**24**	GGTGAGAAAGGAGGG	0.01	3.44 × 10^3^	1.00	0.319	0.325	1.00

^1^ column definitions: F- frequency in sample, β- regression coefficient, STAT- coefficient t-statistic, P- asymptotic *p*-value for t-statistic, EMP1- empirical *p*-value (adaptive), EMP2- corrected empirical *p*-value. ^2^ Frequency of the haplotype allele was calculated based on 220 BeadChips.

**Table 2:** Comparison of CD32 and CD18 read counts in milk and blood derived from RNA-Seq experiments in Holstein.

	Milk										Blood							
		Number of Reads						RPKM				Number of Reads					RPKM	
# ^1^	SRA	Total	1	4	7	9	Sum ^2^	CD32	CD18	R ^3^	SRA	Total	1	4	7	9	CD32	CD18
1	394	27,331,010	1	17	6	176	**200**	27.4	201.2		270	8,584,110	0	5	5	104	36.4	378.6
2	376	19,981,694	5	7	7	205	**225**	31.3	320.6		226	21,522,312	0	2	2	173	5.8	251.2
3	254	18,609,806	0	13	0	219	**232**	21.8	367.7		250	1,177,238	0	0	0	16	0.0	424.7
4	367	16,301,126	3	2	10	220	**235**	28.8	421.8		218	22,950,370	0	13	17	192	40.8	261.4
5	368	20,876,820	0	19	7	209	**235**	38.9	312.8		170	14,416,000	0	17	16	164	71.5	355.5
6	340	21,342,290	10	54	0	173	**237**	93.7	253.3		235	13,338,032	0	26	0	144	60.9	337.4
7	341	25,687,198	4	42	0	203	**249**	56.0	247.0		236	16,838,534	1	26	0	406	50.1	753.5
8	210	14,517,104	2	6	9	235	**252**	36.6	505.9		257	9,046,998	0	0	7	103	27.6	355.8
9	361	23,695,512	0	22	9	247	**279**	42.2	325.7		207	13,028,116	0	15	8	274	55.2	657.2
10	232	17,678,968	0	0	3	306	**309**	5.3	540.9	−0.55	168	23,333,558	0	2	34	122	48.2	163.4
11	188	40,398,796	3	42	8	277	**330**	41.0	214.3	−0.65	214	23,555,514	1	26	20	223	63.7	295.8
12	345	26,493,242	2	40	0	299	**341**	49.5	352.7	−0.67	193	23,755,222	0	51	0	307	67.1	403.9
13	318	41,897,140	0	177	103	68	**348**	208.8	50.7	−0.81	229	6,410,488	0	9	2	59	53.6	287.6
14	321	45,013,510	15	32	32	362	**441**	54.8	251.3	−0.79	230	21,228,336	1	19	19	303	504.9	446.0
15	365	18,179,706	0	7	3	439	**449**	17.2	754.6	−0.73	258	11,983,986	0	9	4	157	33.9	409.4
16	316	30,449,572	0	24	15	410	**449**	40.0	420.8	−0.72	261	3,872,402	0	7	1	61	64.6	492.3
17	243	28,918,832	8	22	12	443	**485**	45.4	478.7	−0.74	217	18,766,692	3	19	11	214	55.0	356.3
18	349	30,480,908	10	88	0	541	**639**	100.5	554.7	−0.61	273	8,150,358	0	12	0	119	46.0	456.3

^1^ Each line presents an individual cow, omitting the SRX2235 prefix from its matching SRA IDs. ^2^ Table is sorted by the sum of counts of reads for CD32 transcript-matching probes 1–8 and, probe 9 matching CD18. ^3^ Correlation was calculated between the preceding CD32 and CD18 RPKM records over a sliding window of 10 lines.

## Supplementary Materials

The following are available online at https://www.mdpi.com/2073-4425/11/8/952/s1, File S1: Dataset (includes ID numbers of cow, sire, dam, paternal and maternal grand sires; liters of milk yield on day of testing; mean SCC recorded for the relevant lactation; health group; bacteriology of the glands; percentages of cells with surface antigens CD18, PMN, CD4, CD8, CD14, BTA3; SNP alleles of BTA3 haplotypes), Table S1: Nucleotide sequences (32 bp probes) used for Megablast search (fasta format), Table S2: Comparison of number of RNA-probe hits and expression levels between *FCGR2*s (CD32) and CD18, in Holstein milk, Table S3: Comparison of number of genomic-probe hits and gene copies between *FCGR2*s (CD32) and control genes.
