# Supplementary material for: Basal Levels of CD18 Antigen Presenting Cells in Cow Milk Associate with Copy Number Variation of Fc Gamma Receptors
Source: Genes (Basel). 2020 Aug 18;11(8):952. doi: 10.3390/genes11080952 (PMC7464846; doi:10.3390/genes11080952)
Supplement: Supplementary file 1 [file genes-11-00952-s001.zip › Tables S1-S3 .pdf]

Table S1 Nucleotide sequences (32 bp probes) used for Megablast search (fasta format).

```
>CD32_Probe1_V1_FCGR2A
TTTAGGGCCGGCAGCAATGACGGTGGGTCCTA
>CD32_Probe2_V2_FCGR2B_bison
TTTAGGGCCGGCAGCAATGACAGTGGGTCCTA
>CD32_Probe3_V3
TTTAGGGCCGGCAGCAACGACGGTGGGTCCTA
>CD32_Probe4_V4_FCGR2B
TTTAGGGCCGGCAGCAACGACAGTGGGTCCTA
>CD32_Probe5_V5
TTTAGGGCCAGCAGCAATGACGGTGGGTCCTA
>CD32_Probe6_V6
TTTAGGGCCAGCAGCAATGACAGTGGGTCCTA
>CD32_Probe7_V7_FCGR2C
TTTAGGGCCAGCAGCAACGACAGTGGGTCCTA
>CD32_Probe8_V8
TTTAGGGCCAGCAGCAACGACGGTGGGTCCTA
>CD18_Probe9
CAGCTGTCCCCACAGGAAGTGACGCTCTACCT
>HOXB7_Probe10_ex1_BTA19
ATGAGTTCATTGTATTATGCGAATGCTTTATT
>HOXA7_Probe11_ex1_BTA4
ATGAGTTCTTCGTATTATGTGAACGCGCTTTT
>SRY_Probe12_ex1_BTY
ATGTTCAGAGTATTGAACGACGATGTTTACAG
>CD32_V4B_FCGR2B
AGGCAACCTCACCACGTGGTTCCATAACGGGA
```

Table S2 Comparison of number of RNA-probe hits and expression levels between FCGR2s (CD32) and CD18, in Holstein milk.

**SRA**	**Sample**	**Total read**	**Probe hits**									**Hit**	***CD32***	***CD18***
		**count**	**1**	**2**	**3**	**4**	**5**	**6**	**7**	**8**	**9**	**sum**	**RPKM**	**RPKM**
SRX2235349	Y10ST0132-W2-Milk-RNA	30,480,908	10	0	0	88	0	0	0	0	541	**639**	100.5	554.7
SRX2235243	213012869-W2-Milk-RNA	28,918,832	8	0	0	22	0	0	12	0	443	**485**	45.4	478.7
SRX2235316	214016531-W2-Milk-RNA	30,449,572	0	0	0	24	0	0	15	0	410	**449**	40.0	420.8
SRX2235365	213012837-W2-Milk-RNA	18,179,706	0	0	0	7	0	0	3	0	439	**449**	17.2	754.6
SRX2235321	214016548-W2-Milk-RNA	45,013,510	15	0	0	32	0	0	32	0	362	**441**	54.8	251.3
SRX2235318	214016543-W2-Milk-RNA	41,897,140	0	0	0	177	0	0	103	0	68	**348**	208.8	50.7
SRX2235345	Y10ST0052-W2-Milk-RNA	26,493,242	2	0	0	40	0	0	0	0	299	**341**	49.5	352.7
SRX2235188	213012829-W2-Milk-RNA	40,398,796	3	0	0	42	0	0	8	0	277	**330**	41.0	214.3
SRX2235232	213012868-W2-Milk-RNA	17,678,968	0	0	0	0	0	0	3	0	306	**309**	5.3	540.9
SRX2235361	211012549-W2-Milk-RNA	23,695,512	0	0	1	22	0	0	9	0	247	**279**	42.2	325.7
SRX2235210	213012834-W2-Milk-RNA	14,517,104	2	0	0	6	0	0	9	0	235	**252**	36.6	505.9
SRX2235341	Y09ST0117-W2-Milk-RNA	25,687,198	4	0	0	42	0	0	0	0	203	**249**	56.0	247.0
SRX2235340	Y09ST0099-W2-Milk-RNA	21,342,290	10	0	0	54	0	0	0	0	173	**237**	93.7	253.3
SRX2235368	213012901-W2-Milk-RNA	20,876,820	0	0	0	19	0	0	7	0	209	**235**	38.9	312.8
SRX2235367	213012871-W2-Milk-RNA	16,301,126	3	0	0	2	0	0	10	0	220	**235**	28.8	421.8
SRX2235254	208000985-W2-Milk-RNA	18,609,806	0	0	0	13	0	0	0	0	219	**232**	21.8	367.7
SRX2235376	214016522-W2-Milk-RNA	19,981,694	5	0	0	7	0	1	7	0	205	**225**	31.3	320.6
SRX2235394	Y09ST0100-W2-Milk-RNA	27,331,010	1	0	0	17	0	0	6	0	176	**200**	27.4	201.2
SRX2235392	Y09ST0076-W2-Milk-RNA	16,939,670	0	0	0	0	0	0	9	0	184	**193**	16.6	339.4
SRX2235362	213012796-W2-Milk-RNA	15,556,506	1	0	0	23	0	0	0	0	163	**187**	48.2	327.4
SRX2235353	210004788-W2-Milk-RNA	34,156,568	5	0	0	55	0	0	1	0	125	**186**	55.8	114.4
SRX2235386	210004820-W2-Milk-RNA	25,894,168	10	0	2	66	0	0	2	0	96	**176**	96.5	115.9
SRX2235348	Y10ST0086-W2-Milk-RNA	13,871,560	0	0	0	32	0	0	0	0	133	**165**	72.1	299.6
SRX2235364	210004794-W2-Milk-RNA	22,742,542	0	0	0	24	0	0	0	0	138	**162**	33.0	189.6
SRX2235381	214016570-W2-Milk-RNA	22,677,018	2	0	0	111	0	0	0	0	47	**160**	155.7	64.8
SRX2235339	Y09ST0054-W2-Milk-RNA	23,314,056	0	0	0	7	0	0	12	0	133	**152**	25.5	178.3
SRX2235199	213012832-W2-Milk-RNA	16,135,480	0	0	0	11	0	0	2	0	139	**152**	25.2	269.2
SRX2235342	209021321-W2-Milk-RNA	12,847,312	1	0	0	31	0	0	1	0	107	**140**	80.3	260.3
SRX2235221	213012838-W2-Milk-RNA	11,794,498	2	0	0	3	0	0	1	0	134	**140**	15.9	355.0
SRX2235177	213012826-W2-Milk-RNA	24,880,104	1	0	0	19	0	0	10	0	106	**136**	37.7	133.1
SRX2235355	210004816-W2-Milk-RNA	10,383,028	2	0	1	22	0	0	0	0	109	**134**	75.2	328.1
SRX2235317	214016533-W2-Milk-RNA	27,837,496	0	0	0	4	0	0	16	0	113	**133**	22.5	126.9
SRX2235373	214016499-W2-Milk-RNA	28,657,822	0	0	0	53	0	0	3	0	74	**130**	61.1	80.7
SRX2235354	210004803-W2-Milk-RNA	16,343,722	3	0	0	14	0	0	11	0	101	**129**	53.5	193.1
SRX2235319	214016546-W2-Milk-RNA	24,965,984	1	0	0	33	0	0	26	0	66	**126**	75.1	82.6
SRX2235301	Y06BO0004-W2-Milk-RNA	6,705,334	2	0	0	15	0	0	0	0	107	**124**	79.2	498.7
SRX2235378	214016540-W2-Milk-RNA	20,432,112	1	0	0	13	0	0	0	0	99	**113**	21.4	151.4
SRX2235331	209021318-W2-Milk-RNA	10,553,652	0	0	0	6	0	0	0	0	104	**110**	17.8	308.0
SRX2235306	Y10ST0126-W2-Milk-RNA	7,716,406	0	0	0	1	0	0	4	0	105	**110**	20.2	425.2
SRX2235305	Y10ST0079-W2-Milk-RNA	8,508,420	2	0	0	10	0	0	3	0	90	**105**	55.1	330.6
SRX2235344	Y10ST0047-W2-Milk-RNA	16,705,320	0	0	0	12	0	0	0	0	91	**103**	22.4	170.2
SRX2235288	214016509-W2-Milk-RNA	16,609,864	0	0	0	6	0	0	1	0	96	**103**	13.2	180.6
SRX2235299	214016523-W2-Milk-RNA	24,236,758	4	0	0	87	0	0	0	0	10	**101**	117.3	12.9
SRX2235277	214016494-W2-Milk-RNA	12,940,436	0	0	0	8	0	0	9	0	84	**101**	41.1	202.9
SRX2235358	210005486-W2-Milk-RNA	7,163,216	0	0	0	3	0	0	3	0	93	**99**	26.2	405.7
SRX2235363	213012818-W2-Milk-RNA	12,392,676	0	0	0	9	0	0	2	0	87	**98**	27.7	219.4
SRX2235396	Y09ST0153-W2-Milk-RNA	13,279,474	0	0	0	40	0	0	0	0	57	**97**	94.1	134.1
SRX2235371	214016492-W2-Milk-RNA	13,705,344	2	0	0	32	0	0	0	0	60	**94**	77.5	136.8
SRX2235315	Y10ST0106-W2-Milk-RNA	12,607,454	0	0	0	4	0	0	2	0	82	**88**	14.9	203.3
SRX2235369	213012919-W2-Milk-RNA	31,039,956	0	0	0	23	0	0	26	0	37	**86**	49.3	37.3
SRX2235304	Y09ST0083-W2-Milk-RNA	7,800,166	4	0	0	2	0	0	0	0	76	**82**	24.0	304.5
SRX2235289	208000940-W2-Milk-RNA	9,973,050	0	0	0	0	0	0	0	0	82	**82**	0.0	256.9
SRX2235395	Y09ST0128-W2-Milk-RNA	16,016,804	0	0	0	11	0	0	18	0	52	**81**	56.6	101.5
SRX2235295	214016489-W2-Milk-RNA	9,471,438	2	0	0	19	0	0	0	0	60	**81**	69.3	198.0
SRX2235393	Y09ST0078-W2-Milk-RNA	9,676,464	0	0	0	11	0	0	0	0	65	**76**	35.5	209.9
SRX2235377	214016527-W2-Milk-RNA	23,508,288	0	0	0	63	0	0	0	0	11	**74**	83.7	14.6
SRX2235310	214016526-W2-Milk-RNA	23,607,534	0	0	0	34	0	0	13	0	25	**72**	62.2	33.1
SRX2235338	Y09ST0026-W2-Milk-RNA	26,595,194	1	0	0	24	0	0	2	0	39	**66**	31.7	45.8
SRX2235293	213012898-W2-Milk-RNA	4,584,264	0	0	0	11	0	0	2	0	49	**62**	88.6	334.0
SRX2235266	213012914-W2-Milk-RNA	12,837,108	0	0	0	9	0	0	9	0	43	**61**	43.8	104.7
SRX2235390	Y04BO0166-W2-Milk-RNA	13,976,318	4	0	0	6	0	0	4	0	47	**61**	31.3	105.1
SRX2235379	214016550-W2-Milk-RNA	24,224,716	0	0	0	13	0	0	27	1	18	**59**	52.9	23.2
SRX2235294	213012904-W2-Milk-RNA	9,372,862	0	0	0	8	0	0	6	0	45	**59**	46.7	150.0
SRX2235291	211012561-W2-Milk-RNA	4,487,086	0	0	0	11	0	0	3	0	45	**59**	97.5	313.4
SRX2235255	213012893-W2-Milk-RNA	11,382,064	2	0	2	17	0	0	0	0	37	**58**	57.7	101.6
SRX2235346	Y10ST0081-W2-Milk-RNA	8,742,160	0	0	0	22	0	0	9	0	26	**57**	110.8	92.9
SRX2235290	208000943-W2-Milk-RNA	7,165,874	0	0	0	3	0	0	0	0	54	**57**	13.1	235.5
SRX2235292	213012889-W2-Milk-RNA	5,177,104	0	0	0	3	0	0	0	0	51	**54**	18.1	307.8
SRX2235356	210004823-W2-Milk-RNA	11,763,706	3	0	0	18	0	0	0	0	32	**53**	55.8	85.0
SRX2235372	214016496-W2-Milk-RNA	25,479,902	0	0	0	24	0	0	25	0	1	**50**	60.1	1.2
SRX2235374	214016508-W2-Milk-RNA	17,872,296	1	0	0	11	0	0	9	0	28	**49**	36.7	49.0
SRX2235296	214016563-W2-Milk-RNA	9,263,740	0	0	0	2	0	0	6	0	40	**48**	27.0	134.9
SRX2235322	214016569-W2-Milk-RNA	22,603,920	0	0	0	30	0	0	0	0	15	**45**	41.5	20.7
SRX2235380	214016555-W2-Milk-RNA	12,594,296	0	0	0	8	0	0	5	0	32	**45**	32.3	79.4
SRX2235360	210005511-W2-Milk-RNA	24,511,974	5	0	0	28	0	0	0	0	11	**44**	42.1	14.0
SRX2235350	Y10ST0136-W2-Milk-RNA	10,947,772	0	0	0	6	0	0	0	0	37	**43**	17.1	105.6
SRX2235285	Y10ST0036-W2-Milk-RNA	4,577,696	1	0	0	10	0	0	0	0	30	**41**	75.1	204.8
SRX2235366	213012845-W2-Milk-RNA	10,152,012	0	0	0	22	0	0	0	0	17	**39**	67.7	52.3
SRX2235347	Y10ST0082-W2-Milk-RNA	17,935,162	0	0	0	30	0	0	1	0	5	**36**	54.0	8.7
SRX2235279	213012766-W2-Milk-RNA	2,394,778	0	0	0	9	0	0	0	0	26	**35**	117.4	339.3
SRX2235357	210004824-W2-Milk-RNA	8,029,858	0	0	0	4	0	0	10	0	20	**34**	54.5	77.8
SRX2235280	213012816-W2-Milk-RNA	3,969,810	0	0	0	3	0	0	0	0	29	**32**	23.6	228.3
SRX2235391	Y09ST0062-W2-Milk-RNA	14,806,576	3	0	1	15	0	0	0	0	11	**30**	40.1	23.2
SRX2235283	214016518-W2-Milk-RNA	3,076,422	0	0	0	2	0	0	0	0	28	**30**	20.3	284.4
SRX2235352	210004789-W2-Milk-RNA	10,517,488	0	0	0	11	0	0	0	0	15	**26**	32.7	44.6
SRX2235359	210005510-W2-Milk-RNA	12,409,976	0	0	0	10	0	0	4	0	7	**21**	35.3	17.6
SRX2235278	211012537-W2-Milk-RNA	4,175,764	0	0	0	3	0	0	0	0	18	**21**	22.5	134.7
SRX2235302	Y09ST0018-W2-Milk-RNA	5,427,298	0	0	0	5	0	0	1	0	14	**20**	34.5	80.6
SRX2235375	210004818-W2-Milk-RNA	4,449,212	2	0	0	3	0	0	0	0	12	**17**	35.1	84.3
SRX2235351	208000916-W2-Milk-RNA	3,285,532	0	0	0	1	0	0	2	0	12	**15**	28.5	114.1
SRX2235286	Y10ST0065-W2-Milk-RNA	1,023,366	0	0	0	1	0	0	0	0	13	**14**	30.5	397.0
SRX2235303	Y09ST0037-W2-Milk-RNA	7,543,566	0	0	0	3	0	0	2	0	7	**12**	20.7	29.0
SRX2235274	207016597-W2-Milk-RNA	2,642,858	0	0	0	5	0	0	2	0	0	**7**	82.8	0.0
SRX2235308	210004817-W2-Milk-RNA	515,606	0	0	0	1	0	0	0	0	6	**7**	60.6	363.6
SRX2235370	213012936-W2-Milk-RNA	2,122,970	0	0	0	0	0	0	0	0	6	**6**	0.0	88.3
SRX2235275	208001021-W2-Milk-RNA	977,394	0	0	0	0	0	0	0	0	6	**6**	0.0	191.8
SRX2235281	213012827-W2-Milk-RNA	862,206	0	0	0	0	0	0	0	0	4	**4**	0.0	145.0
SRX2235343	Y10ST0038-W2-Milk-RNA	59,914	0	0	0	0	0	0	0	0	0	**0**	0.0	0.0
SRX2235287	Y10ST0125-W2-Milk-RNA	1,769,292	0	0	0	0	0	0	0	0	0	**0**	0.0	0.0
SRX2235282	214016493-W2-Milk-RNA	513,466	0	0	0	0	0	0	0	0	0	**0**	0.0	0.0

Table S3 Comparison of number of genomic-probe hits and gene copies between FCGR2s (CD32) and control genes.

SRA	Total read	Sex	Breed	Probe hits											Allele	*FCGR2A*	*FCGR2B*	*FCGR2C*	*CD32*	*HOXB7*	*HOXA7*	*SRY*	*CD32*
	count			1	2	3	4	5	6	7	8	9	10	11	coverage	V1	V4	V7	total	probe9	probe10	probe11	copy #
**SRX5388796**	470,474,464	male	Holstein	13	0	0	7	0	0	10	0	23	21	4	12	1.1	0.6	0.8	2.5	1.9	1.8	0.3	3.1
**SRX5388795**	588,981,714	male	Holstein	33	0	0	8	0	0	12	0	35	33	11	15	2.2	0.5	0.8	3.6	2.4	2.2	0.7	3.4
**SRX5388794**	489,874,890	male	Holstein	0	0	0	20	0	0	16	0	23	10	8	12	0.0	1.6	1.3	2.9	1.9	0.8	0.6	4.4
**SRX5388793**	587,942,612	male	Holstein	9	0	0	25	0	0	14	0	23	18	9	15	0.6	1.7	0.9	3.2	1.6	1.2	0.6	4.8
**SRX5388792**	567,993,322	male	Holstein	0	0	0	28	0	0	9	0	23	25	6	14	0.0	2.0	0.6	2.6	1.6	1.7	0.4	3.4
**SRX5388791**	628,575,808	male	Holstein	21	0	0	25	0	0	0	0	19	24	11	16	1.3	1.6	0.0	2.9	1.2	1.5	0.7	4.3
**SRX5388790**	424,180,494	male	Holstein	14	0	0	10	0	0	0	0	18	20	6	11	1.3	0.9	0.0	2.2	1.7	1.9	0.6	2.7
**SRX5388788**	576,077,866	male	Holstein	16	0	0	17	0	0	9	0	24	17	5	14	1.1	1.2	0.6	2.9	1.7	1.2	0.3	4.6
**SRX5388783**	555,070,320	male	Holstein	22	0	0	11	0	0	5	0	13	23	12	14	1.6	0.8	0.4	2.7	0.9	1.6	0.9	4.0
**SRX5388782**	535,278,424	male	Holstein	9	0	0	17	0	0	0	0	26	17	6	13	0.7	1.3	0.0	1.9	1.9	1.3	0.4	2.7
**SRX5388772**	331,842,454	female	Holstein	3	0	0	4	0	0	3	0	4	5	3	8	0.4	0.5	0.4	1.2	0.5	0.6	0.4	4.2
**SRX5388760**	333,947,112	male	Holstein	5	0	0	5	0	0	0	0	8	4	2	8	0.6	0.6	0.0	1.2	1.0	0.5	0.2	3.6
**SRX5388759**	351,401,114	female	Holstein	7	0	0	0	0	0	2	0	9	6	0	9	0.8	0.0	0.2	1.0	1.0	0.7	0.0	2.4
**SRX5388722**	621,669,354	male	Holstein	22	0	0	9	0	0	23	0	21	35	3	16	1.4	0.6	1.5	3.5	1.3	2.2	0.2	4.6
**SRX5388721**	466,874,314	male	Holstein	5	0	0	12	0	0	8	0	8	22	13	12	0.4	1.0	0.7	2.1	0.7	1.9	1.1	2.9
**SRX5388720**	421,586,302	male	Holstein	0	0	0	13	0	0	14	0	8	15	6	11	0.0	1.2	1.3	2.5	0.8	1.4	0.6	4.7
**SRX5388719**	564,266,702	male	Holstein	22	0	0	15	0	0	0	0	17	34	13	14	1.5	1.1	0.0	2.6	1.2	2.4	0.9	2.9
**SRX5388718**	618,128,856	male	Holstein	30	0	0	11	0	0	11	0	27	18	10	16	1.9	0.7	0.7	3.3	1.7	1.2	0.6	4.7
**SRX5388717**	580,249,376	male	Holstein	14	0	0	23	0	0	7	0	21	14	16	15	1.0	1.6	0.5	3.0	1.4	1.0	1.1	4.3
**SRX5388716**	571,900,668	male	Holstein	0	0	0	25	0	0	20	0	16	14	7	14	0.0	1.7	1.4	3.1	1.1	1.0	0.5	6.1
**SRX5388798**	333,869,172	ND	Angus	6	0	0	6	0	0	0	0	4	3	1	8	0.7	0.7	0.0	1.4	0.5	0.4	0.1	7.5
**SRX5388780**	493,208,872	male	Angus	15	0	0	20	0	0	0	0	10	20	11	12	1.2	1.6	0.0	2.8	0.8	1.6	0.9	4.3
**SRX5388779**	388,734,538	male	Angus	8	0	0	4	0	0	7	0	8	16	5	10	0.8	0.4	0.7	1.9	0.8	1.6	0.5	3.3
**SRX5388778**	451,330,720	male	Angus	2	0	0	4	0	0	2	0	8	8	2	11	0.2	0.4	0.2	0.7	0.7	0.7	0.2	2.2
**SRX5388634**	229,153,424	male	Hereford	0	0	0	11	0	0	0	0	2	4	1	6	0.0	1.9	0.0	1.9	0.3	0.7	0.2	7.9
**SRX5388768**	134,268,496	male	Hereford	1	0	0	3	0	0	0	0	3	3	1	3	0.3	0.9	0.0	1.2	0.9	0.9	0.3	2.9
**SRX5388764**	206,098,750	male	Hereford	4	0	0	4	0	0	0	0	1	4	1	5	0.8	0.8	0.0	1.5	0.2	0.8	0.2	6.7
**SRX5388417**	507,699,238	male	Hereford	18	0	0	20	0	0	0	0	16	25	10	13	1.4	1.6	0.0	3.0	1.3	2.0	0.8	3.7
**SRX5388706**	345,045,756	male	Charolaise	2	0	0	4	0	0	4	0	5	11	5	9	0.2	0.5	0.5	1.2	0.6	1.3	0.6	2.4
**SRX5388705**	371,643,412	male	Charolaise	10	0	0	14	0	0	0	0	5	5	3	9	1.1	1.5	0.0	2.6	0.5	0.5	0.3	9.2
**SRX5388641**	454,126,982	male	Charolaise	4	0	0	10	0	0	0	0	10	5	2	11	0.3	0.9	0.0	1.2	0.9	0.4	0.2	4.1
**SRX5388510**	427,558,928	male	Charolaise	0	0	0	16	0	0	0	0	9	10	6	11	0.0	1.5	0.0	1.5	0.8	0.9	0.6	3.2
**SRX5388642**	402,309,450	male	Limousine	3	0	0	6	0	0	0	0	5	3	4	10	0.3	0.6	0.0	0.9	0.5	0.3	0.4	3.8
**SRX5388640**	434,740,428	male	Limousine	0	0	0	18	0	0	0	0	8	8	3	11	0.0	1.6	0.0	1.6	0.7	0.7	0.3	4.7
**SRX5388639**	366,943,370	male	Limousine	1	0	0	6	0	0	0	0	10	4	2	9	0.1	0.6	0.0	0.8	1.1	0.4	0.2	2.2
**SRX5388637**	418,123,230	male	Limousine	0	0	0	9	0	0	0	0	9	10	1	11	0.0	0.9	0.0	0.9	0.9	1.0	0.1	2.3
**SRX7427485**	571,200,404	male	Simmental	15	0	0	6	0	0	9	0	27	17	10	14	1.0	0.4	0.6	2.1	1.9	1.2	0.7	2.8
**SRX7427463**	607,416,724	male	Simmental	5	0	0	16	0	0	7	0	21	21	17	15	0.3	1.0	0.5	1.8	1.4	1.4	1.1	2.4
**SRX7427462**	587,475,052	female	Simmental	11	0	0	14	0	0	5	0	23	27	0	15	0.7	0.9	0.3	2.0	1.6	1.8	0.0	2.4
**SRX5388697**	493,971,372	male	Simmental	0	0	0	10	0	0	0	0	14	6	2	12	0.0	0.8	0.0	0.8	1.1	0.5	0.2	2.3
